# Establishing a Model for Evaluating Chicken Coccidiosis Resistance Based on Principal Component Analysis

**DOI:** 10.3390/ani9110926

**Published:** 2019-11-06

**Authors:** Wenbin Zou, Hailiang Yu, Xiaohui Wang, Guojun Dai, Mingming Sun, Genxi Zhang, Tao Zhang, Huiqiang Shi, Kaizhou Xie, Jinyu Wang

**Affiliations:** 1 College of Animal Science and Technology, Yangzhou University, Yangzhou 225009, China; wenbinzou1216@163.com (W.Z.); hailiangyu122514@163.com (H.Y.); wxh9409161412@126.com (X.W.); zgx1588@126.com (G.Z.); zhangt@yzu.edu.cn (T.Z.); yzxkz168@163.com (K.X.); jywang@yzu.edu.cn (J.W.); 2 Key Lab for Animal Genetics, Breeding and Reproduction and Molecular Design of Jiangsu Province, Yangzhou 225009, China; 3 Department of Animal Science, University of Wyoming, Laramie, Wyoming, WY 82072, USA; 4 Center for Cardiovascular Research and Integrative Medicine, University of Wyoming, Laramie, Wyoming, WY 82072, USA; 5 Jiangsu Jinghai Poultry Industry Group Co., Ltd., Nantong 226103, China; 13921648510@163.com

**Keywords:** coccidiosis resistance parameter, principal component analysis, evaluation model, selection index

## Abstract

**Simple Summary:**

Avian coccidiosis, an infectious disease caused by seven species of *Eimeria* that can infect a bird’s digestive tract and significantly retard its growth, is a serious economic disease for chickens. Many studies have demonstrated that host resistance to coccidiosis related to genetic variations can be improved by selective breeding. The parameters for evaluation of resistance to coccidiosis could be objective indicators, such as body weight gain and cecal lesion score, or biochemical indices, such as immune factors or cytokines in the plasma or serum. The aim of the study is to establish an optimal comprehensive evaluation model including a resistance index that can be detected in live chickens (slaughter traits cannot be selected in breeding) based on principal component analysis. The value of individual chickens calculated with the optimal evaluation model is associated with the cecum lesion score; the larger the value, the stronger the resistance to coccidiosis. This illustrated that the optimal model is effective in coccidiosis resistance selection.

**Abstract:**

To establish a coccidiosis resistance evaluation model for chicken selection, the different parameters were compared between infected and control Jinghai yellow chickens. Validation parameters were selected for principal component analysis (PCA), and an optimal comprehensive evaluation model was selected based on the significance of a correlation coefficient between coccidiosis resistance parameters and principal component functions. The following six different parameters were identified: body weight gain 3–5 days post infection and catalase (CAT), superoxide dismutase (SOD), glutathione peroxidase (GSH-Px), malondialdehyde (MDA) and γ-interferon (IFN-γ) concentrations on the eight day post inoculation. Six principal components and one accumulated contribution of up to 80% of the evaluation models were established by PCA. The results showed that the first model was significantly or highly significantly related to nine resistance parameters (*p* < 0.01 or *p* < 0.05), especially to cecal lesions (*p* < 0.01). The remaining models were related to only 2–3 parameters (*p* < 0.01 or *p* < 0.05) and not to cecal lesions (*p* > 0.05). The values calculated by the optimal model (first model) were significantly negatively correlated with cecal lesion performance; the larger the value, the more resistant to coccidiosis. The model fi1 = −0.636 zxi1 + 0.311 zxi2 + 0.801 zxi3 − 0.046 zxi4 − 0.076 zxi5 + 0.588 zxi6 might be the best comprehensive selection index model for chicken coccidiosis resistance selection.

## 1. Introduction

Avian coccidiosis is a type of infectious parasitosis. One of the most pathogenic species is *Eimeria tenella* (*E. tenella*), which can invade chicken cecum epithelial cells [1]. The hazard of clinical coccidiosis is highest in the early stage (weeks 1–3) [2]. The morbidity and mortality of chickens aged ~10–30 days and ~35–60 days could reach 80% [3]. A financial loss of approximately 3 billion dollars in the poultry industry is caused by coccidiosis worldwide every year, more than 74% of which is due to retarded growth and reduced feed conversion ratio in chickens and the rest is accounted for by the increase in spending for treatments with drugs [4,5,6]. Anticoccidial drugs are mainly used to control avian coccidiosis; however, chemotherapy can lead to the problem of coccidiosis resistance to drugs, which can reduce the efficacy of the drugs. The extensive use of drugs will increase feeding costs and lead to concerns about drug residues [7]. Although vaccination can be used to prevent coccidiosis, some problems remain to be solved, such as effectiveness and safety [8]. Therefore, to solve this problem, coccidiosis resistance in chickens should be studied to develop genetic selection methods to cultivate coccidiosis-resistant breeds or strains for poultry production [9]. To date, the evaluation method used in the selection of coccidiosis resistance in chickens has not been mentioned in the existing literature. The parameters for evaluating resistance can be divided into two classes: objective indicators, such as body weight gain and cecal lesion score, and biochemical indices, such as immune factors or cytokines in the plasma or serum [10,11,12,13]. SOD, CAT, GSH-Px and MDA constitute an antioxidant system in the body. CAT is an oxidoreductase capable of decomposing hydrogen peroxide, GSH-Px can work with SOD to remove active oxidative free radicals, MDA can degrade lipid peroxidation products [14,15]. Some studies have used antioxidant markers and gamma interferon as indicators of resistance to coccidiosis [16,17]. Moreover, neither chicken selection by means of resistance parameters nor a comprehensive selection index model has been reported. Many studies have demonstrated that variations of resistance to coccidiosis exist in inbred and outbred chicken lines, and the results of these studies have set a reliable foundation for coccidiosis-resistant chicken breeding [18,19,20,21,22]. In this study, Jinghai yellow chickens were used as the experimental material to compare significant differences in the indicators, and the indicators with significant differences were used for principal component analysis. A comprehensive evaluation model was selected based on the significant correlation coefficients between each of the coccidiosis resistance parameters, especially the objective indicators, and principal component functions.

## 2. Materials and Methods

### 2.1. Animals

Sixty-six one-day-old Jinghai yellow chicks obtained from Jiangsu Jinghai Poultry Industry Group Co., Ltd. (Nantong, China), were randomly divided into the following 2 groups: 44 chicks were in the infection group, and the remaining chicks were in the control group.

### 2.2. Coccidiosis Challenge

The chicks were raised in specific pathogen-free housing until they were 30 days old, then transferred into individual wire cages, given food and water ad libitum, and raised with the same standard raising protocol. The chicks were kept in cages sterilized with a gasoline torch flame without specific pathogens, the feed contains no coccidia or anticoccidials, and the diet meets the chicks’ nutritional needs [23]. The infected group was orally inoculated with 2.5 × 10^4^ sporulated *E. tenella* oocysts (obtained from the Department of Parasitology at the College of Veterinary Medicine of Yangzhou University) at 30 days of age, and the control group was orally inoculated with the same volume of saline. All protocols for animal sample collection were approved by the Animal Welfare Committee of Yangzhou University (permit number: SYXK (Su) IACUC 2012-0029), and all efforts were made to minimize the suffering of the chickens.

### 2.3. Detection of Resistance-Associated Parameters

Body weight gain: All chickens were weighed on days 0, 3, 5, and 8 post inoculation (PI). The body weight gains during four periods, BWG0–3, BWG3–5, BWG5–8, and BWG0–8, were calculated from days 0 to 3, 3 to 5, 5 to 8, and 0–8 PI, respectively. Cecal lesion score: The cecal lesion score was assessed at day 8 PI by using the method previously described by Johnson and Reid [24]. To eliminate bias, the lesion scores (ranging from 0 to 4, with 5 levels) for each individual were observed by only one person [25]. Biochemical indices: Blood samples were collected from each bird in heparinized tubes on day 8 PI and centrifuged at 1000× *g* for 15 min to recover the plasma. The plasma samples were stored at −20 °C until further analysis. The biochemical indices detected in plasma were nitric oxide (NO), catalase (CAT), superoxide dismutase (SOD), glutathione peroxidase (GSH-Px), malondialdehyde aldehyde (MDA), interleukin-2 (IL-2), interleukin-16 (IL-16), interleukin-17 (IL-17), γ-interferon (IFN-γ), and β-carotene (β-C) concentrations. Biotinylated double-antibody sandwich enzyme-linked immunosorbent assay (ELISA) was used to measure those biochemical indices (resistant parameters) according to the ELISA kit instructions. ELISA kits were purchased from Shanghai Yueyan Biotechnology Co., Ltd., China.

### 2.4. Statistical Analysis

#### 2.4.1. Principal Component Analysis

Data were analyzed with the PASW Statistics 18.0 software (SPSS Inc., Armonk, NY, USA. 2009). For resistance parameter selection, an independent two-sample t test was conducted to compare the significance of the resistance parameters between the infection and control groups; only the significantly different indicators between the two groups were selected as valid resistance parameters to perform principal component analysis (PCA). For PCA, the valid selected original data were standardized by using the formula: ${\mathrm{zx}}_{\mathrm{ij}}{=(x}_{\mathrm{ij}}\;{-\;\bar{x}}_{j}{)/s}_{j}$ and calculated by using the descriptive program of the PASW software, where ${\bar{x}}_{j}$, S_j_ and zx_ij_ are the mean, standard deviation and standardized value, respectively, of the jth valid resistance parameters; i is the ith individual (i = 1, 2, 3, ……, n); and j is the jth valid resistance parameter (j = 1, 2, 3, …, p). The standardized values were subjected to PCA through the Factor program in the PASW software to obtain the eigenvalues ($\lambda_{1}\geq \lambda_{2}\geq \cdots \geq \lambda_{p}\geq 0$) of the zxij correlation matrix, the contribution rate ($\lambda_{i}/\sum_{i=1}^{p}\lambda_{i}$) and the cumulative contribution rate ($\sum_{i=1}^{k}\lambda_{i}/\sum_{i=1}^{p}\lambda_{i}$) of the eigenvalues. Generally, as the cumulative contribution rate is greater than 80–90% (as the case may be), the former k principal component (PCo) would be involved in the comprehensive evaluation model for coccidiosis resistance. Supposing that fij (PCoj) represents the expression function of the ith individual jth principal component, the linear combination for principal components can be denoted as follows:

$$\left\{\begin{array}{c} f_{i1}{=a}_{11}{\mathrm{zx}}_{i1}{+a}_{12}{\mathrm{zx}}_{i2}+\cdots {+a}_{1p}{\mathrm{zx}}_{\mathrm{ip}}\\ f_{i2}{=a}_{21}{\mathrm{zx}}_{i1}{+a}_{22}{\mathrm{zx}}_{i2}+\cdots {+a}_{2p}{\mathrm{zx}}_{\mathrm{ip}}\\ \cdots \\ f_{\mathrm{ip}}{=a}_{p1}{\mathrm{zx}}_{i1}{+a}_{p2}{\mathrm{zx}}_{i2}+\cdots {+a}_{\mathrm{pp}}{\mathrm{zx}}_{\mathrm{ip}} \end{array}\right.$$

where (*a_ij_*) is the eigenvector matrix. The comprehensive evaluation model (comprehensive PCo) can be calculated by the following equation:

$$f_{i}{=(\lambda }_{1}f_{i1}{+\lambda }_{2}f_{i2}+\cdots {+\lambda }_{k}f_{\mathrm{ik}}/\overset{k}{\underset{j=1}{\sum }}\lambda_{j}$$

where *f_i_* represents the comprehensive evaluation value of the *i*th individual.

#### 2.4.2. Correlation Analysis

The correlation between PCo (including comprehensive PCo) and the standardized resistance parameters was calculated by Pearson’s correlation, and the correlation between PCo and the standardized cecal lesion score was presented by Spearman analysis.

## 3. Results

### 3.1. The Selection of Valid Resistant Parameters

According to the comparison of the resistance traits (see Table 1 and Table 2), six of 14 candidate indictors (except for the cecal lesion score, which is a slaughter trait that cannot be selected in breeding) were significantly different between the two groups: body weight gain in 3–5 days and concentrations of CAT, SOD, GSH-Px, MDA, and IFN-γ in the plasma at 8 days PI after treatment with *E. tenella*.

### 3.2. Contribution Rate and the Cumulative Contribution Rate of the Eigenvalues

The comprehensive evaluation model for coccidiosis resistance could be constructed based on the six standardized indicators chosen from Table 1 and Table 2. According to Table 3 (PCA results), the cumulative rate contribution of the top four principal components is almost 80%. Therefore, these four principal components can explain all the variation involved in the original variables and could be used to establish the comprehensive coccidiosis resistance evaluation model. 

### 3.3. Establishing the Coccidiosis Resistance Evaluation Model

The products of the eigenvectors (Table 4) and the corresponding standardized valid resistance parameter values are summed to calculate the expression function of the principal component (Table 5). The absolute value of the coefficients of the function could reflect the effect of each indicator on the principal component, and the positive or negative sign could reflect the positive or negative correlations of the indicators with the PCo.

Because the cumulative contribution rate of 1–4 PCo is up to 80%, the comprehensive PCo equation is as follows: $f_{i}{=(\lambda }_{1}f_{i1}{+\lambda }_{2}f_{i2}+\cdots {+\lambda }_{4}f_{i4}/\sum_{j=1}^{4}\lambda_{j}$ i.e., $f_{i}=(24.956f_{i1}+19.905f_{i2}+18.714f_{i3}+16.058f_{i4})/79.633$, then fi1~fi4 is plugged into the equation, and the comprehensive principal component model is obtained (as shown in Table 5).

### 3.4. The Selection of the Optimal PCo

**Figure 1:**
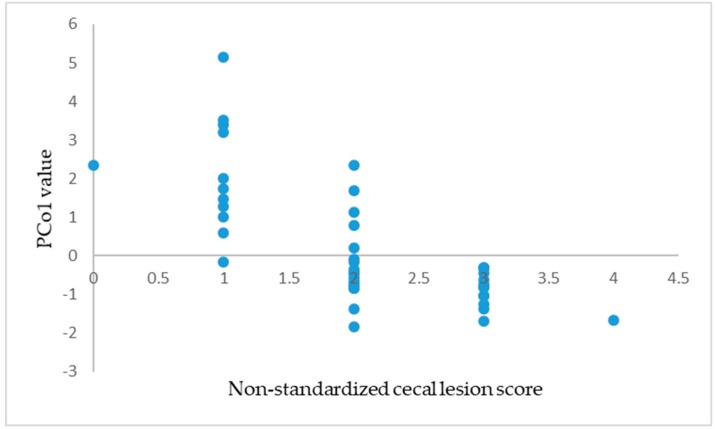
The scatter plot of non-standardized cecal lesion scores with PCo1.

According to Table 6, PCo1 had a highly significant correlation with the cecal lesion score, the concentrations of NO, SOD, IFN-γ, and body weight gain in the 0–3, ~3–5, and ~5–8 day intervals (*p* < 0.01) and a significant correlation with the concentration of CAT and body weight gain during the ~0–8 day interval (*p* < 0.05). PCo2 had a highly significant correlation with the concentrations of CAT and MDA (*p* < 0.01) and a significant correlation with GSH-Px (*p* < 0.05). PCo3 had a highly significant correlation with the concentrations of GSH-Px and IFN-γ (*p* < 0.01). PCo4 had a highly significant correlation with the concentration of CAT and the body weight gain during the 3–5 day intervals (*p* < 0.01). PCo5 had a highly significant correlation with the concentrations of MDA and IFN-γ and the body weight gain in the 3–5 day interval (*p* < 0.01). PCo6 had a highly significant correlation with the concentration of SOD (*p* < 0.01) and a significant correlation with the concentration of IFN-γ (*p* < 0.05). However, the comprehensive PCo merely had a highly significant correlation with the concentration of SOD (*p* < 0.01) and a significant correlation with the concentration of GSH-Px (*p* < 0.05). Figure 1 is the scatter plot of the non-standardized cecal lesion score with PCo1. The standardized cecal lesion scores are five discrete levels. Briefly, the two variables are negatively correlated.

In conclusion, PCo1 could be the optimized evaluation model for coccidiosis resistance because it had a highly significant correlation with most indicators, especially with the cecal lesion score and body weight gain, which could directly reflect the level of infection among chickens.

## 4. Discussion

### 4.1. The Selection of Resistance Parameters

Various resistance parameters were used to estimate coccidiosis resistance in chickens, and the ordinary resistance indicators were the anticoccidial index (ACI), reduction of lesion scores (RLS), relative oocyst production (ROP), percentage of optimum anticoccidial activity (POAA) and fecal scoring [11,12,26,27]. Body weight gain, fecal oocyst shedding, and the concentrations of NO, β-C and IFN-γ were used by Zhu et al. [10], and the feed conversion rate was additionally used by Williams and Catchpole [28]. The concentrations of SOD, MDA, CAT and GSH-Px were used by Li et al. [29] and Georgieva et al. [14]. Zhang et al. [30] suggested that plasma NO, IFN-γ, SOD, MDA, and IL-17 can be used as markers of resistance to *E. tenella*. Some of the indicators, such as body weight gain and the plasma indicators, could be measured without slaughter, while others, such as the lesion scores, could be obtained only by dissecting the chicks, as the slaughtered chicken has no breeding value. Some of the indicators were objective evaluation indices, such as body weight; however, others, such as the number of spores in the feces, had greater sampling error and could not be used in some small-scale tests. In brief, the objective evaluation indices and some indicators with small sampling errors such as body weight gain (BWG), concentrations of CAT, SOD, GSH-Px, MDA, and IFN-γ are more suitable to be employed in breeding for resistance to disease, and the six indicators selected in this study have these attributes.

### 4.2. The Advantage of PCA for the Selective Breeding of Coccidiosis Resistance

Zhu et al. [10] proposed an infection index (II) formula by calculating indicators related to coccidian resistance. The II formula can be expressed as follows: II = Σ(C × (individual value of a parameter − mean of the parameter in the group)/SD). An individual II is equal to the sum of the difference between an individual parameter value and the mean of the group divided by the SD and multiplied by a factor. The factor C is +1 and −1 for the parameters negatively and positively correlated to BWG, respectively. The results showed that the infection index (II) may be a better parameter for evaluating individual genetic resistance against coccidial infection. In this study, PCA was first proposed by Pearson [31] to reduce data dimensionality. First, PCA is an effective data analysis tool to identify and express patterns in data and then highlight the data similarities and differences. Second, PCA can compress data by reducing the dimensionality of a dataset that consists of a large number of interrelated variables without much loss of information [32]. The data compression is performed by transforming the original data into a new set of variables, the new principal components, which are uncorrelated with each other. Through the use of PCA, the dimensionality of data is reduced, and multicollinearity is eliminated [33]. As a result, PCA is an effective method for minimizing the limitations of previous studies, i.e., multicollinearity, subjectivity and high computation requirement. To date, no papers have been published on the application of PCA to solve the coccidiosis resistance selection problem. Therefore, PCA was employed in this study. Fourteen candidate indicators were filtered according to a number of previous studies, and six of them were chosen as crucial indicators applied in the PCA, which could improve the accuracy of the final model.

### 4.3. The Selection and Evaluation of the Optimized Model

The lesion score and the body weight gain could directly mirror the levels of infection among chickens; however, the remaining six principal components had a significant correlation with several indicators that were less correlated with cecal lesion score and body weight gain. Therefore, they could not represent the coccidiosis resistance of chickens. Ye [34] suggested that the correlation between the principal component and the original variables should be considered to determine the number of principal components. Fu [35] argued that the choice of optimizing principal components was neither necessarily ranked by the proportion percent nor reached 85% of the accumulation. Therefore, it is accurate and reasonable to choose PCo1 as the optimized model.

According to the expression functions of PCo1, BWG3–5 and the concentrations of CAT, SOD and IFN-γ had higher coefficient in the equation, which suggested that the four indicators could also indirectly reveal the level of coccidiosis resistance. According to Table 6, PCo1 had a highly significant negative correlation with the cecal lesion score and a significant positive correlation with body weight gain in <~0–8 days (*p* < 0.05), which illustrated that the larger the PCo1 value was, the better the coccidiosis resistance exhibited by the chicken. It seemed paradoxical that PCo1 had a highly significant negative correlation with body weight gain in ~3–5 days, this is because the swelling of the intestine causes the chickens that are severely infected during this period to be heavier than the healthy ones, as the *E. tenella* was generally the most severe period for the Jinghai yellow chicken ~3–5 days after the infection. 

The infected group individual index which was calculated according to PCo1 optimized model illustrated that the individual with an index greater than 2.01 had almost no significant difference from the control group individuals. As the selection index decreased, the cecum lesion increased. However, if the index was less than −1.55, there would be a blood core in the cecum. The lower the index, the larger the blood core is, and the more susceptible individuals are to coccidiosis.

In this study, the selected optimal evaluation model was established on the chickens raised on wire and challenged with a specific infection dose, which are different from litter or floor, whether the model is still applicable needs to be verified, additionally, as the optimal evaluation model was established on 44 individuals, it needs to be tested in a larger population to determine if the model is to be considered useful in future trials. 

## 5. Conclusions

There were six valid resistant parameters that had significant differences between the infected and control groups among the selected 14 potential parameters: body weight gain on the 3rd–5th day PI and the CAT, GSH-Px, MDA, SOD and IFN-γ concentrations on day 8 PI.

The accumulated contribution of six principal components reached 80%, and comprehensive resistance evaluation models were established by PCA. The optimized model of fi1 = −0.636 zxi1 + 0.311 zxi2 + 0.801 zxi3 − 0.046 zxi4 − 0.076 zxi5 + 0.588 zxi6 might be the best coccidiosis resistance comprehensive selection index model and could be used for chicken breeding for resistance selection.

The largest and smallest four values calculated from the optimized model were in accordance with the cecal lesion performance.

## Figures and Tables

**Table 1:** Initial body weight and body weight gain.

Traits (g)	Control Group (*n* = 22)	Infected Group (*n* = 44)	*t*-Value	Probability
IBW * (0 dPI)	215.79 ± 6.02	215.42 ± 4.69		
IBW (3 dPI)	242.91 ± 6.65	241.98 ± 5.45		
IBW (5 dPI)	252.15 ± 6.89	240.96 ± 5.43		
IBW (8 dPI)	266.88 ± 8.12	255.69 ± 5.65		
BWG ** 0–3	27.13 ± 1.21	26.55 ± 0.75	0.155	0.877
BWG3–5	9.25 ± 0.44	−1.03 ± 0.10	3.062	0.004 **
BWG5–8	14.75 ± 2.33	14.72 ± 2.81	0.032	0.995
BWG0–8	51.00 ± 2.41	40.30 ± 3.07	1.970	0.054

Note: * IBW indicates initial body weight, ** BWG indicates body weight gain.

**Table 2:** Coccidiosis resistance trait differences between the infected and control groups on day 8 PI (post inoculation).

Resistance Traits	Control Group (*n* = 22)	Infected Group (*n* = 44)	*t-*Value	Probability
Nitric oxide (NO) (µmol/L)	55.49 ± 1.23	54.10 ± 0.80	0.853	0.398
catalase (CAT) (U/L)	54.26 ± 2.32	63.28 ± 1.66	2.744	0.008 **
superoxide dismutase (SOD) (U/L)	106.87 ± 3.29	120.60 ± 2.85	2.558	0.014 *
glutathione peroxidase (GSH-Px) (U/L)	396.31 ± 11.88	455.72 ± 11.01	2.902	0.005 **
malondialdehyde aldehyde (MDA) (mmol/L)	4.75 ± 0.20	5.86 ± 0.15	3.789	0.001 **
γ-interferon (IFN-γ) (ng/L)	38.88 ± 1.08	35.89 ± 0.69	2.100	0.041 *
β-carotene (β-C) (μmol/L)	60.56 ± 5.53	65.49 ± 4.04	0.620	0.538
interleukin-2 (IL-2) (ng/L)	35.31 ± 1.14	33.05 ± 0.87	1.335	0.188
interleukin-16 (IL-16) (ng/L)	47.88 ± 2.39	52.84 ± 1.19	1.842	0.071
interleukin-17 (IL-17) (ng/L)	40.78 ± 0.96	38.70 ± 1.22	0.960	0.341
Cecal lesion score	0.00 ± 0.00	1.98 ± 0.11	10.975	0.000 **

Note: * indicates a significant difference (*p* < 0.05), and ** indicates a highly significant < difference (*p* < 0.01). Values are denoted as the mean ± standard errors.

**Table 3:** Principal component analysis (PCA) results based on the six coccidiosis resistance traits (*n* = 44).

No. of PCo	Eigenvalue (λ*_i_*)	Contribution Rate (%)	Cumulative Rate (%)
PCo 1	1.497	24.956	24.956
PCo 2	1.194	19.905	44.861
PCo 3	1.123	18.714	63.575
PCo 4	0.963	16.058	79.633
PCo 5	0.740	12.335	91.968
PCo 6	0.482	8.032	100.000

Note: PCo indicates principal components.

**Table 4:** Eigenvectors of the 6 principal resistance trait components.

Standardized Traits	Eigenvectors of Principal Components					
	${a1}_{j}\;$	${a2}_{j}$	${a3}_{j}$	${a4}_{j}$	${a5}_{j}$	${a6}_{j}$
BWG3–5 (zx1)	−0.636	0.296	0.177	0.417	0.498	0.234
CAT (zx2)	0.311	−0.478	0.069	0.792	−0.004	−0.207
SOD (zx3)	0.801	0.277	−0.183	0.187	−0.005	0.461
GSH-Px (zx4)	−0.046	−0.349	0.857	−0.082	−0.246	0.272
MDA (zx5)	−0.076	0.779	0.250	0.290	−0.461	−0.166
IFN-γ (zx6)	0.588	0.269	0.505	−0.192	0.469	−0.266

**Table 5:** Expression functions of the principal component model.

No. of PCo	Expression Functions of Each Principal Component Model
PCo 1	$f_{i1}=-0.636{\mathrm{zx}}_{i1}+0.311{\mathrm{zx}}_{i2}+0.801{\mathrm{zx}}_{i3}-0.046{\mathrm{zx}}_{i4}-0.076{\mathrm{zx}}_{i5}+0.588{\mathrm{zx}}_{i6}\;$
PCo 2	$f_{i2}=0.296{\mathrm{zx}}_{i1}-0.478{\mathrm{zx}}_{i2}+0.277{\mathrm{zx}}_{i3}-0.349{\mathrm{zx}}_{i4}+0.779{\mathrm{zx}}_{i5}+0.269{\mathrm{zx}}_{i6}\;$
PCo 3	$f_{i3}=0.177{\mathrm{zx}}_{i1}+0.069{\mathrm{zx}}_{i2}-0.183{\mathrm{zx}}_{i3}+0.857{\mathrm{zx}}_{i4}+0.250{\mathrm{zx}}_{i5}+0.505{\mathrm{zx}}_{i6}\;$
PCo 4	$f_{i4}=0.417{\mathrm{zx}}_{i1}+0.792{\mathrm{zx}}_{i2}+0.187{\mathrm{zx}}_{i3}-0.082{\mathrm{zx}}_{i4}+0.290{\mathrm{zx}}_{i5}-0.192{\mathrm{zx}}_{i6}\;$
PCo 5	$f_{i5}=0.498zx_{i1}+0.004{\mathrm{zx}}_{i2}-0.005{\mathrm{zx}}_{i3}-0.246{\mathrm{zx}}_{i4}-0.461{\mathrm{zx}}_{i5}+0.469{\mathrm{zx}}_{i6}\;$
PCo 6	$f_{i6}=0.234zx_{i1}\;-0.207{207\mathrm{zx}}_{i2}+0.461{\mathrm{zx}}_{i3}+0.272{\mathrm{zx}}_{i4}-0.166{\mathrm{zx}}_{i5}-0.266{\mathrm{zx}}_{i6}\;$
Comprehensive PCo	$f_{i}=0.001zx_{i1}\;+0.166{\mathrm{zx}}_{i2}+0.378{\mathrm{zx}}_{i3}+0.082{\mathrm{zx}}_{i4}+0.292{\mathrm{zx}}_{i5}+0.329{\mathrm{zx}}_{i6}\;$

**Table 6:** The correlation between principal components and standardizing the coccidiosis resistance traits (*n* = 44).

Standardized Traits	PCo 1	PCo 2	PCo 3	PCo 4	PCo 5	PCo 6	Comprehensive PCo
Cecal lesion score ^▲^	−0.539 **	0.075	0.036	0.036	−0.138	−0.029	−0.368
BWG0–3	0.510 **	−0.200	−0.024	−0.151	−0.291	−0.032	0.142
BWG3–5	−0.635 **	0.237	0.191	0.415 **	0.468 **	0.205	−0.021
BWG5–8	0.426 **	−0.037	−0.176	−0.193	−0.115	−0.034	0.022
BWG0–8	0.340 *	0.096	−0.014	−0.048	0.156	0.072	0.097
NO	0.512 **	−0.011	0.302	−0.240	0.006	0.016	0.261
CAT	0.326 *	−0.464 **	0.012	0.756 **	−0.001	−0.185	−0.225
SOD	0.776 **	0.171	−0.192	0.247	0.061	0.459 **	0.552 **
GSH-Px	−0.047	−0.371 *	0.784 **	−0.153	−0.281	0.267	0.332 *
MDA	−0.076	0.779 **	0.250	0.290	−0.461 **	−0.166	0.109
IFN-γ	0.521 **	0.222	0.513 **	−0.196	0.445 **	−0.353 *	−0.065
β-C	−0.001	−0.089	0.013	0.163	0.263	−0.209	−0.223
IL-2	−0.065	0.049	−0.069	0.004	−0.069	−0.274	−0.302
IL-16	−0.107	−0.189	0.007	0.160	−0.153	0.074	−0.086
IL-17	0.218	−0.127	−0.039	−0.292	−0.154	−0.150	−0.011

Note: ^▲^ indicates the Spearman correlation coefficients. * indicates a significant difference (p < 0.05), and ** indicates a highly significant < difference (p < 0.01).
